# Biodegradation pattern and tissue integration of native and cross-linked porcine collagen soft tissue augmentation matrices – an experimental study in the rat

**DOI:** 10.1186/1746-160X-10-10

**Published:** 2014-03-27

**Authors:** Daniel Rothamel, Marcel Benner, Tim Fienitz, Arndt Happe, Matthias Kreppel, Hans-Joachim Nickenig, Joachim E Zöller

**Affiliations:** 1 Department of Oral and Maxillofacial Plastic Surgery, University of Cologne, Kerpener Str. 62, 50937 Cologne, Germany

**Keywords:** Collagen, Soft tissue augmentation, Bone regeneration, Biodegradation, Cross-linking, Native type I and III collagen

## Abstract

**Introduction:**

Within the last decades, collagen types I and III have been established as a sufficient biomaterial for GBR and GTR procedures. They might also be an adequate matrix for soft tissue augmentations. However, collagen materials differ significantly regarding resorption time, biodegradation pattern and the invasion of inflammatory cells.

The aim of the present study was to compare the biodegradation and tissue integration of native, differently processed and cross-linked collagen scaffolds in rats.

**Methods:**

Four experimental porcine collagen matrices of 1.0 mm thickness, developed for soft tissue augmentation procedures, were tested. Based on the same native dermal Type I and III collagen, native (ND, Mucoderm® prototype), specifically defatted (DD), ethylene dioxide cross-linked (ECL) and dehydrothermally cross-linked (DCL) dermis collagen (AAP/Botiss Biomaterials, Berlin, Germany) were evaluated. Two specimens of 1 × 1 cm were fixed around a non-absorbable spacer using non-absorbable sutures. After rehydration, specimens (N = 8) were randomly allocated in unconnected subcutaneous pouches on the back of 40 Wistar rats. Rats were divided into five groups (1, 2, 4, 8 and 12 weeks), including eight animals each. After each period, eight rats were sacrificed and explanted specimens were prepared for histological analysis. The following parameters were evaluated: membrane thickness as a sign of biodegradation and volume stability, cell ingrowth, vascularization, tissue integration and foreign body reaction.

**Results:**

Biodegradation pattern of the non cross-linked collagen scaffolds differed only slightly in terms of presence of inflammatory cells and cell invasion into the matrix. In terms of biodegradation, ECL displayed a considerable slower resorption than ND, DCL and DD. Chemical cross-linking using ethylene dioxide showed a significant higher invasion of inflammatory cells.

**Conclusion:**

Within the limits of the present study it was concluded that the processing techniques influenced the collagen properties in a different intensity. Dehydrothermal cross-linking and special defatting did not notably change the biodegradation pattern, whereas cross-linking using ethylene dioxide led to significant higher volume stability of the matrix. However, ECL showed an increased inflammatory response and compromised tissue integration. Therefore, ethylene dioxide seems to be not suitable for stabilization of collagen matrices for soft tissue augmentation procedures.

## Introduction

In periodontology and implantology bone and soft tissue augmentation procedures are often necessary to achieve aesthetic and functional results. Regarding bone augmentation Guided Bone and Tissue Regeneration (GBR/GTR) have been established as a comparable alternative to the gold standard, i.e. grafting of autogenous bone [1-5]. The concept of GBR/GTR procedures include the use of bone substitute materials and covering the grafting site with an absorbable membrane. This membrane separates cells with regenerative potential, like osteoblasts and desmodontal fibroblasts, from rapidly proliferating epithelial and connective tissue cells. Different membrane materials have been investigated and collagen has proven to be a promising substance for GBR procedures. As native collagen shows a lack of immunogenicity [6], collagen can be harvested from different donor species and be transferred to humans without immunological response. Postlethwaite et al. [7] pointed out that the advantages of collagen lie in its haemostatic function, chemotactic properties to attract periodontal ligament fibroblasts and gingival fibroblasts as well as semi permeability and support of nutrient transfer. Furthermore, collagen plays an important role in coagulum formation and is a major component of the periodontal connective tissue [8-10]. It also seemed to surpass other materials in design criteria for GBR/GTR such as biocompatibility, tissue integration, cell-occlusivity, volume stability and easy use in the clinical routine [11]. Moreover, native collagen type I/III showed good biocompatibility in vitro and in vivo [12] as well as fast vascularization and revitalization [13,14].

In terms of soft tissue augmentation the use of soft tissue grafts is an accepted routine in implantology and periodontal surgery not only in order to decrease or neutralize pain deriving from soft tissue loss but also because of functional and aesthetic aspects [15-18]. As a result of soft tissue thickening the translucency of restorative materials or discoloured devital teeth and the occurrence of recessions are reduced [16]. Soft tissue augmentation procedures are usually performed by harvesting either autologous free gingival [19] or connective tissue grafts [20]. These procedures are often accompanied by an additional donor site pain [21] and complications like bleeding due to damage of the branches of the palatine artery, necrosis of the mucosa and hyp- or anaesthesia [18]. The quantity and quality of the transplants is individually different and naturally limited [22].

To avoid the aforementioned donor site morbidity and being able to enhance soft tissue regardless of individual prerequisites collagen matrices can also be used for soft tissue augmentations procedures. In contrast to the autogenous grafts, collagen matrices are unlimitedly available and can be produced with a constant quality. In a clinical study performed by Sanz et al. [23] the use of collagen matrices to enhance the width of keratinized gingiva was compared with free connective tissue grafts. Clinical outcome was comparable for both groups. However, patients who were treated by collagen matrices suffered significantly less pain and the total time of surgery was reduced, since no autogenous grafts had to be harvested.

Collagen matrices that are used for soft tissue augmentations have to show adequate volume stability in order to allow enough time for cells to invade into the collagen matrix and to build new soft tissue. In line with the GTR/GBR membranes, rapid biodegradation might be also a disadvantage. Tatakis et al. [24] marked the fast biodegradation caused by the enzymatic activity (collagenase) of macrophages and polymorphonuclear leukocytes as the greatest demerit of native collagen [24]. However, resorption time of collagen can be extended by cross-linking of the collagen fibres using physical, thermic or chemical procedures. Previous studies investigated differently cross-linked GTR/GBR membranes and found substantial differences regarding biodegradation, biocompatibility and angiogenesis. In this context cross-linking using glutaraldehyde decreased the biocompatibility whereas enzymatic cross-linking negatively reduced the tissue integration and biodegradation pattern. The degree of chemical cross-linking correlated with higher volume stability, but caused severe inflammatory reactions [25]. Moreover, a higher inflammatory response was found when collagen scaffolds were not sufficiently defatted.

The aim of the present study was to investigate new kinds of collagen scaffolds stabilization, i.e. ethylene dioxide and dehydrothermal cross-linking as well as a special defatting protocol including acetone treatment, and to compare these techniques to native type I/III collagen.

## Materials and methods

### Animals

Forty albino rats of the Wistar strain (age 3 + - 0.5 months, weight 350 +/- 21 g) were used in the present study. Animal selection, management, and surgery protocol were approved by the Animal Care and Use Committee of the local government of Dusseldorf (LANUV, reference number 87-51.04.2010.A139), Germany. The animals were divided into five groups (1, 2, 4, 8, 12 weeks), including eight rats each.

### Membranes examined

**Figure 1:**
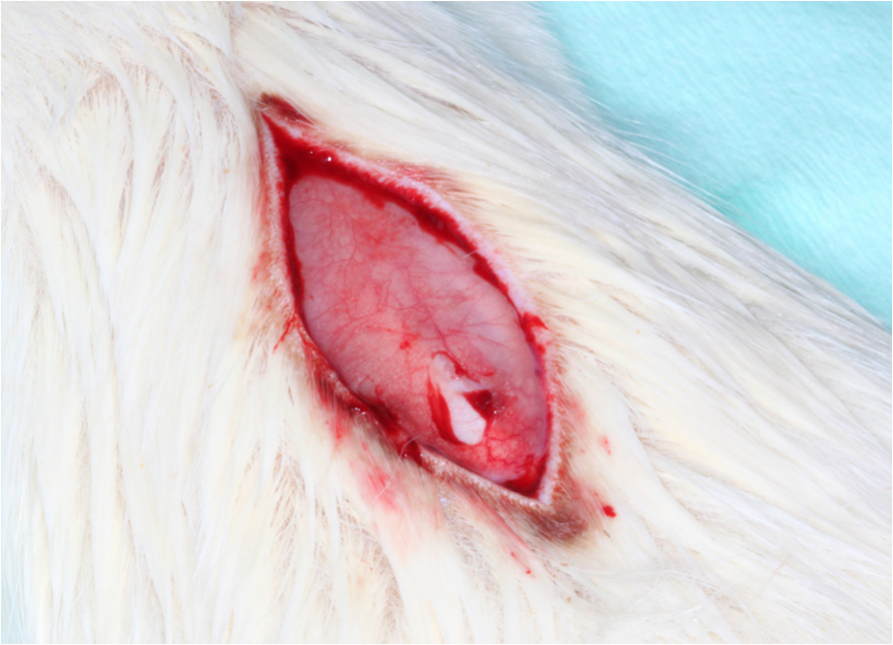
Paravertebral incision for graft implantation.

Four experimental porcine collagen matrices of 1.0 mm thickness, developed for soft tissue augmentation procedures, were tested. Based on the same native dermal Type I and III collagen tissue, native (ND, Mucoderm® Prototype), specifically defatted (DD), ethylene dioxide cross-linked (ECL) and dehydrothermally cross-linked (DCL) dermis collagen (AAP/Botiss Biomaterials, Berlin, Germany) were evaluated. Two specimens of 1 × 1 cm were fixed around a non-absorbable polycarbonate spacer (Isopore®, Millipore Corporate, Billerica, MA, USA) using non-absorbable sutures (Seralon®, Resorba, Nuremberg, Germany) for easier retrieval after longer healing periods (Figure 1). The specimens were circumferentially sutured to avoid an ingrowth of the surrounding tissue. After rehydration in 0.9% sterile saline solution (Braun, Melsungen, Germany), specimens (N = 8) were randomly allocated in unconnected subcutaneous pouches separated surgically on the back of 40 Wistar rats.

### Surgical procedure

**Figure 2:**
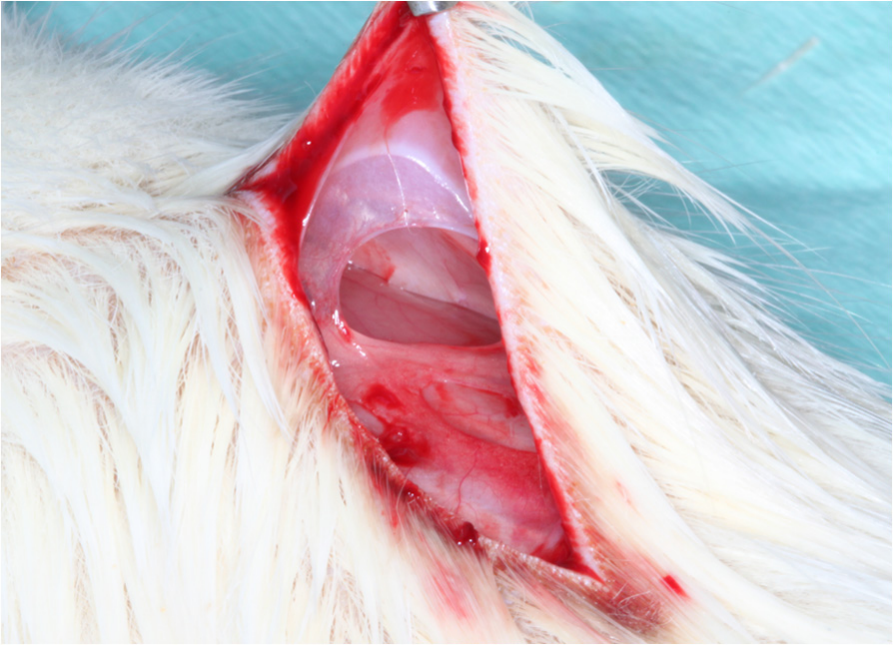
Subcutaneous preparation of four unconnected pouches.

**Figure 3:**
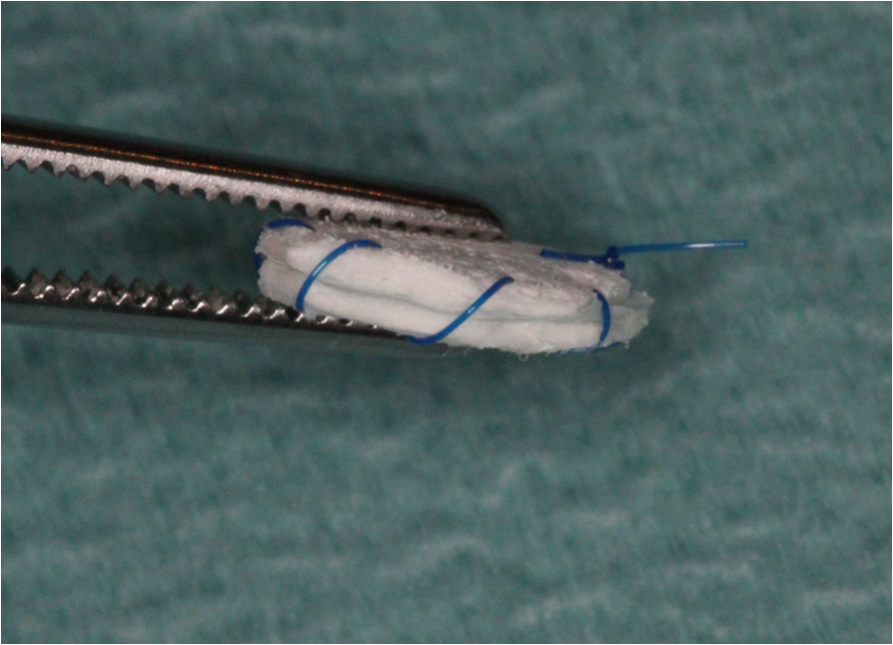
Collagen matrices were marked and fixed around a polycarbonate spacer using a non-resorbable polyester suture.

**Figure 4:**
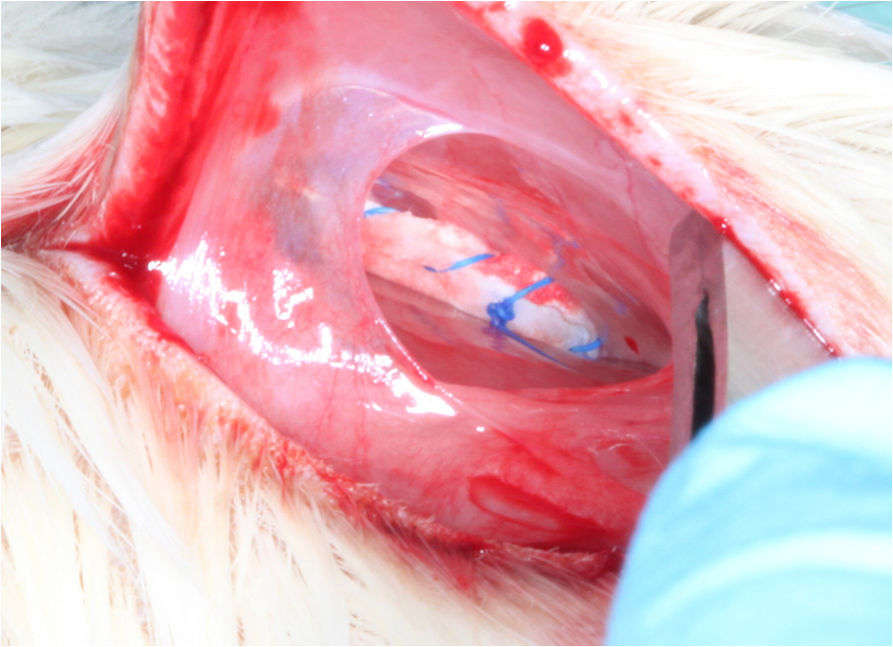
Specimens were rehydrated and carefully allocated into subcutaneous pockets.

**Figure 5:**
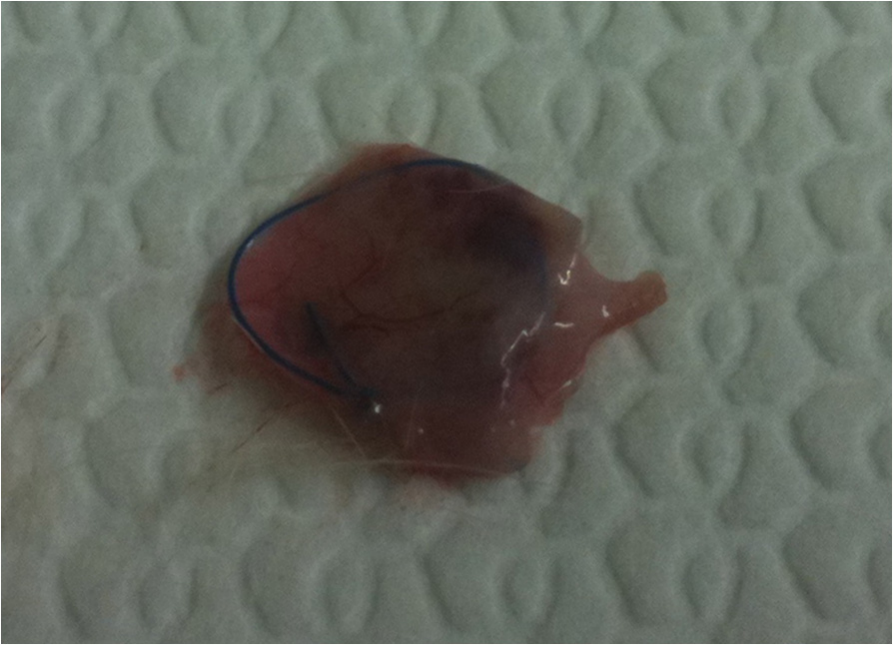
Native dermal collagen after 4 weeks of healing, revealing good tissue integration and macroscopic ingrowth of blood vessels.

The animals were anesthetized by intraperitoneal injection of 9 mg/kg ketamine 10% (Ketanest®, Pfizer GmbH, Karlsruhe, Germany) and 5 mg/kg xylazine 2% (Sedaxylan®, Pfizer GmbH). Following disinfection of the back with polyvidone iodine (Betaisodona®, Mundipharma, Limburg a.d. Lahn, Germany), a skin incision was made exactly paramedian along the vertebral column (Figure 2) followed by the separation of four unconnected subcutaneous pouches (Figure 3). The membranes were randomly allocated in the resulting 160 pouches (Figure 4). Primary wound closure was achieved using horizontal mattress 3.0 Vicryl sutures (Resorba, Nurnberg, Germany). During the experiment, the animals were fed *ad libitum* with standard laboratory food pellets. Animals were sacrificed in a carbon dioxide euthanasia chamber after 1, 2, 4, 8, and 12 weeks. Residues of the membranes were removed with the surrounding connective tissue (Figure 5) and fixed in 10% formalin.

### Histomorphometry

An experienced research associate, blinded to the specific experimental conditions, performed the histomorphometrical analysis and microscopic examination. All specimens were embedded in paraffin. Four histological sections were systemically and randomly cut from each specimen. The resulting serial sections of 7 μm thickness were stained with Goldner Trichrome stain, respectively HE stain. For image acquisition a colour CCD camera (ColorView III, Olympus, Hamburg, Germany) was mounted on a binocular light microscope (Olympus BX50, Olympus). Digital images were evaluated using an imaging program (Cell D, Soft Imaging System, Muenster, Germany). Before the histomorphometrical analysis, a calibration procedure was initiated for the image analysis software, revealing that repeated measurements of n = 12 different sections were similar at >95% level.

The thickness of the membrane body was measured linearly at 12 fields selected at random. Additionally, the following parameters were evaluated descriptively: vascularization of the membrane body, tissue integration and foreign body reaction (i.e. presence of multinucleated giant cells). Cell invasion was classified in five categories and graphically processed as a box plot figure. Histological specimens that showed cell invasion only in the outer third of the collagen scaffold were classified into the first category. Accordingly, category 2 and 3 showed cell invasion up to the second third or complete invasion of the collagen scaffold. Category 4 was assigned to homogenous spreading of cells within the collagen body, whereas category 5 represented complete biodegradation of the membranes.

### Statistical analysis

A statistical software (SPSS 22.0, SPSS Inc., Chicago, IL, USA) was used for the statistical analysis. Mean values and standard deviations were calculated for each group regarding membrane thickness. Analysis of variance (ANOVA) and post hoc testing by Bonferroni's correction for multiple comparisons were used for comparisons within groups. Results were considered statistically significant at P < 0.05.

## Results

### Postoperative healing

The postoperative healing was uneventful in all rats. No complications were observed, including infection, bleeding, allergic reactions or dehiscences.

### Macroscopic analysis

Harvested residues of specimens revealed good tissue integration for all groups. After 1, 2 and 4 weeks matrices were embedded in an inflammation-free layer of subcutaneous tissue, revealing small blood vessels within the matrix and along the surface of the collagen (Figure 5). After 8 and 12 weeks, EDC revealed no macroscopic change of matrix thickness, whereas ND, DD and DCL showed significant reduction of the collagen.

### Histomorphometrical analysis

**Figure 6:**
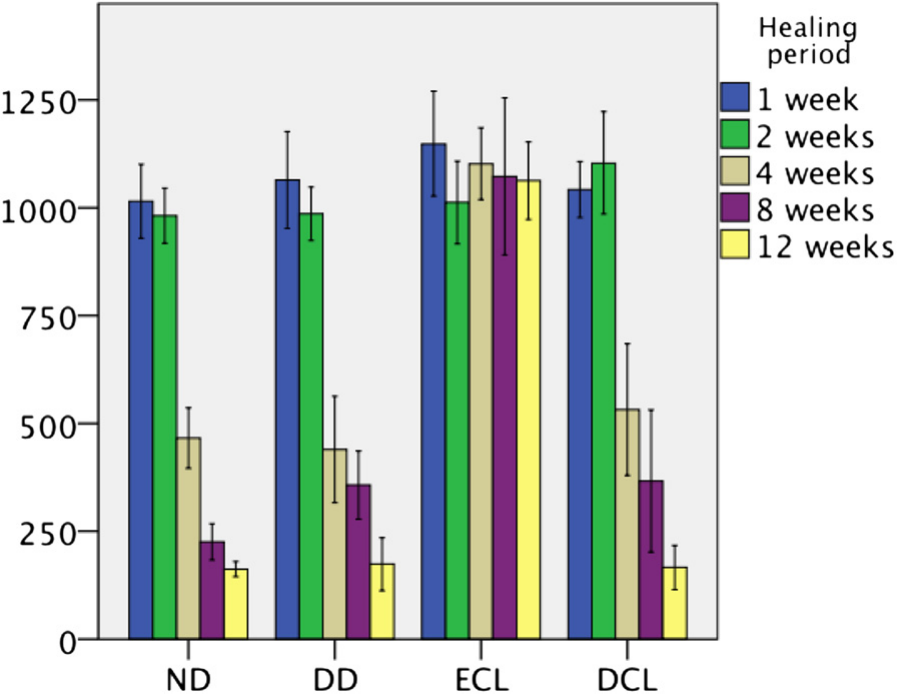
Bars representing means and standard deviations (in nm) of thickness of different collagen matrices after 1, 2, 4, 8 and 12 weeks.

Thickness of matrix bodies for each group at different healing periods is presented in Figure 6. Histomorphometrical analysis revealed that scaffold thickness of all tested scaffolds showed no considerable reduction two weeks following implantation in each group (P > 0.05 respectively). Histomorphometrical analysis failed to demonstrate a reduction of scaffold thickness of ECL during the entire study period of twelve weeks (P > 0.05). ND, DD and DCL showed significant changes of scaffold thickness after four weeks following implantation (P < 0.001 respectively).

**Figure 7:**
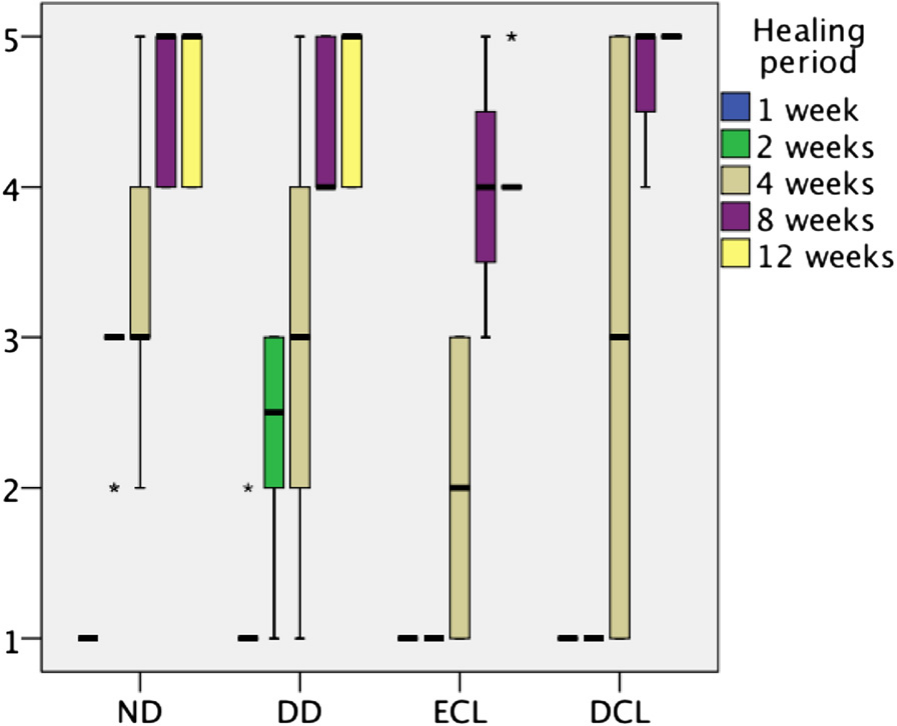
Boxplots representing cell invasion and matrix organization of different collagen matrices (1: < 1/3, 2: < 2/3, 3: < 3/3 of matrix thickness, 4: homogenous organization, 5: complete biodegradation).

Cell invasion and biodegradation values are represented in boxplots due to their non-parametric characteristics (Figure 7). After one week, all matrices revealed cell invasion less than the first third of the matrix. ND and DD showed faster invasion of cells after 2 and 4 weeks, whereas ECL and DCL started to being organized more than the first third earliest after four weeks. ND, DD and DCL displayed complete biodegradation after 12 weeks, whereas ECL revealed a homogenous organisation and no signs of matrix collapse at this time.

### Histological analysis

#### One-week specimens

One week following implantation, three of the scaffolds (ND, DD, DCL) did not show substantial differences regarding the membrane structure. These membranes presented a porous structure with smaller and larger interconnected spaces. ECL presented a slightly denser structure. All four scaffolds displayed a physiological post-traumatic inflammatory reaction in the tissue neighbouring the external surface, the most prominent cells being neutrophil granulocytes and macrophages. Some ND specimens demonstrated a slight vascularization of the superficial portion of the scaffold.

#### Two-week specimens

**Figure 8:**
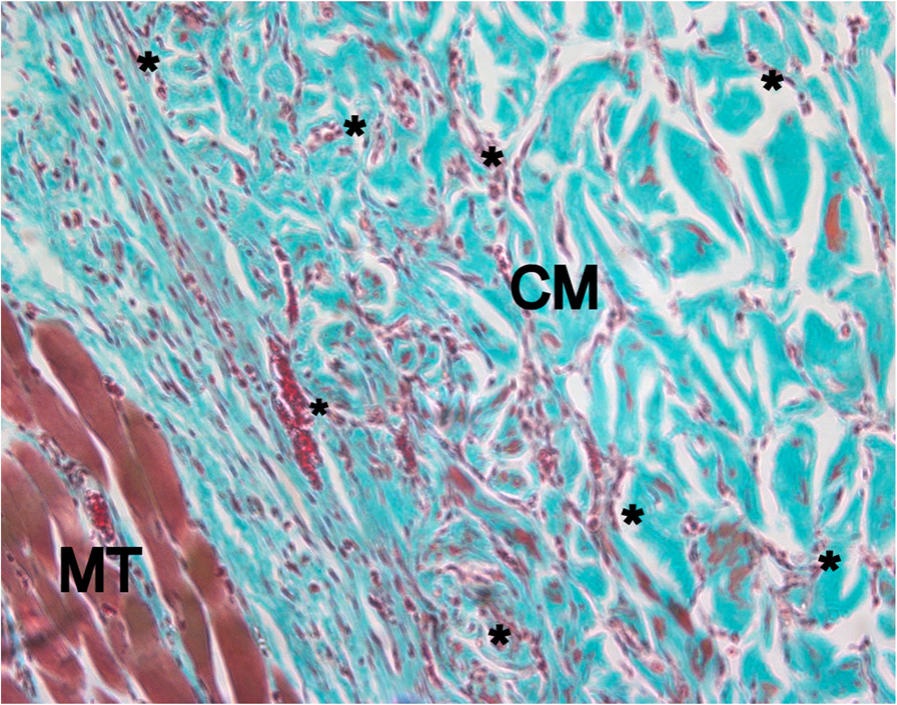
**Native dermal collagen (ND) shows excellent tissue integration and initial cell invasion after 2 weeks healing period (Masson Goldner, orig. magn. 200×).** CM: collagen matrix, MT: muscle tissue, *: blood vessel.

**Figure 9:**
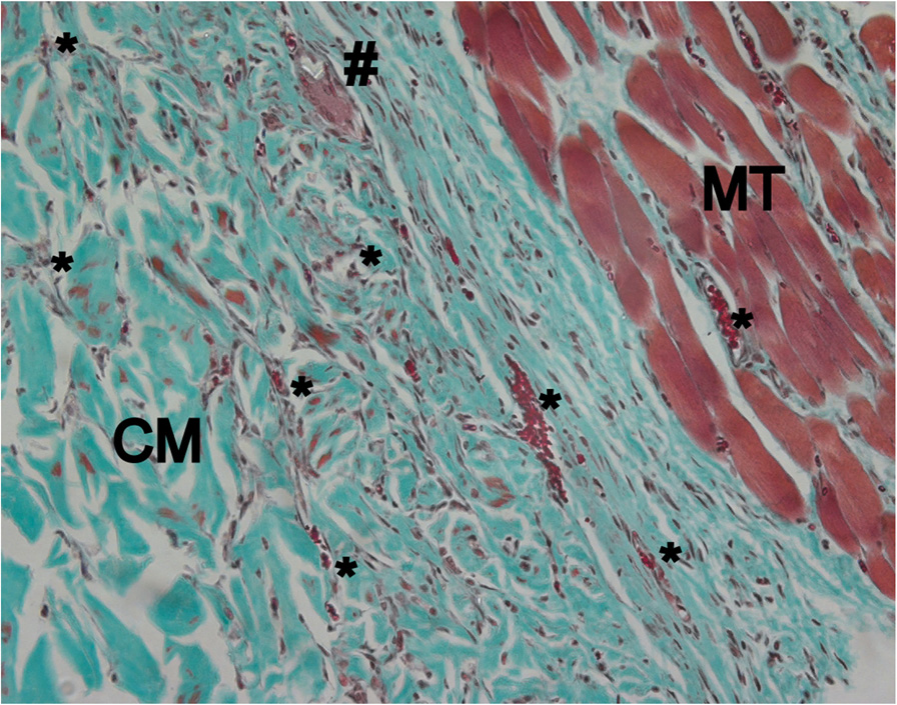
**Single invasion of a multinucleated giant cell in a dehydrothermally cross-linked matrix after 2 weeks healing period (Masson Goldner, orig. magn. 200×).** CM: collagen matrix, MT: muscle tissue, *: blood vessel, #: multinucleated giant cell.

After two weeks, large areas of ND and DD were invaded by blood vessels and scattered neutrophil granulocytes and macrophages (Figure 8). Mononucleated cells invading the outer parts of the collagen scaffolds could be detected in increasing areas also of DCL, partly showing single multinucleated cells in between the collagen strains (Figure 9). Blood vessels ingrowth from the surrounding connective tissues could be also detected. ECL presented only superficial cell invasion, less tissue integration and almost no blood vessel ingrowth into the dense collagen scaffold.

#### Four-week specimens

After four weeks, histological analysis demonstrated a homogenous cell invasion of ND and DD. Most of these scaffolds were almost completely biodegraded and organized by newly formed connective tissue. For DCL a biodegradation of 50% could be noted. ECL scaffolds did not show any changes in thickness or collagen structure, but cell invasion could be detected in the outer thirds. Regarding neo-angiogenesis ND and DD appeared completely vascularized and also most parts of DCL displayed clear signs of vascularization. The appearance of inflammatory cells was limited to isolated macrophages and granulocytes for ND, DD and ECL, whereas DCL demonstrated a significantly higher foreign body reaction, with high numbers of multinuclear giant cells even reaching the inner surface of the scaffold.

#### Eight-week specimens

**Figure 10:**
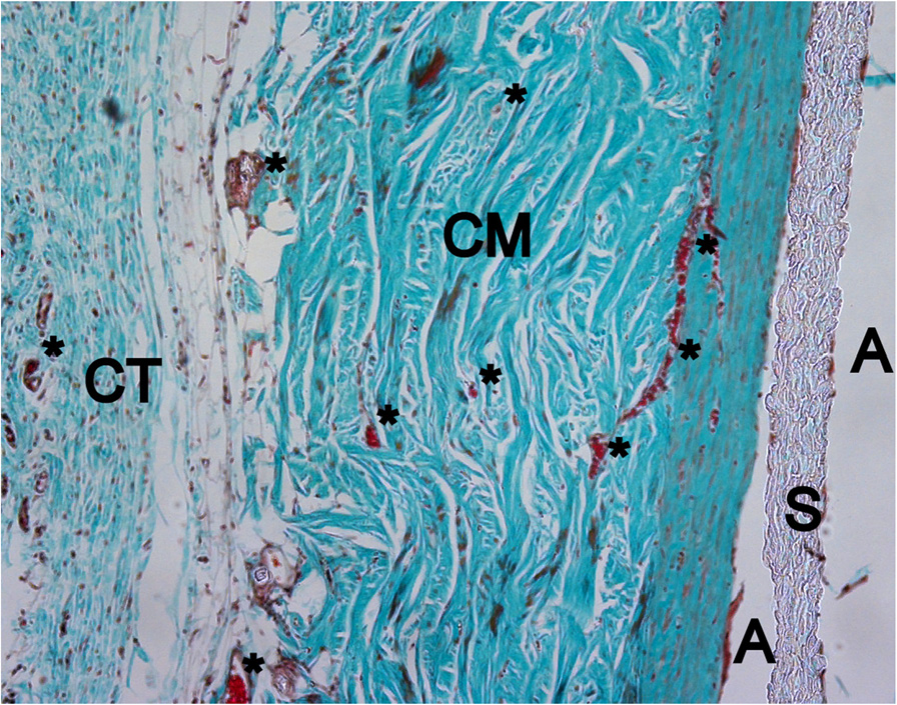
**Advanced biodegradation and no foreign body reaction on DD after 8 weeks healing period (Masson Goldner, orig. magn. 100×).** CM: collagen matrix, MT: muscle tissue, *: blood vessel, CT: connective tissue, S: spacer, A: artefact.

Eight weeks following implantation ND, DD and DCL were completely organized and almost entirely replaced by newly formed connective tissue (Figure 10). The ECL scaffold could still be identified almost with the same matrix thickness as implanted, showing homogenous cell invasion and blood vessel ingrowth reaching the inner surface of the matrix. However, a high concentration of multinucleated giant cells were visible around the individual collagen strains, representing a strong foreign body reaction.

#### Twelve-week specimens

**Figure 11:**
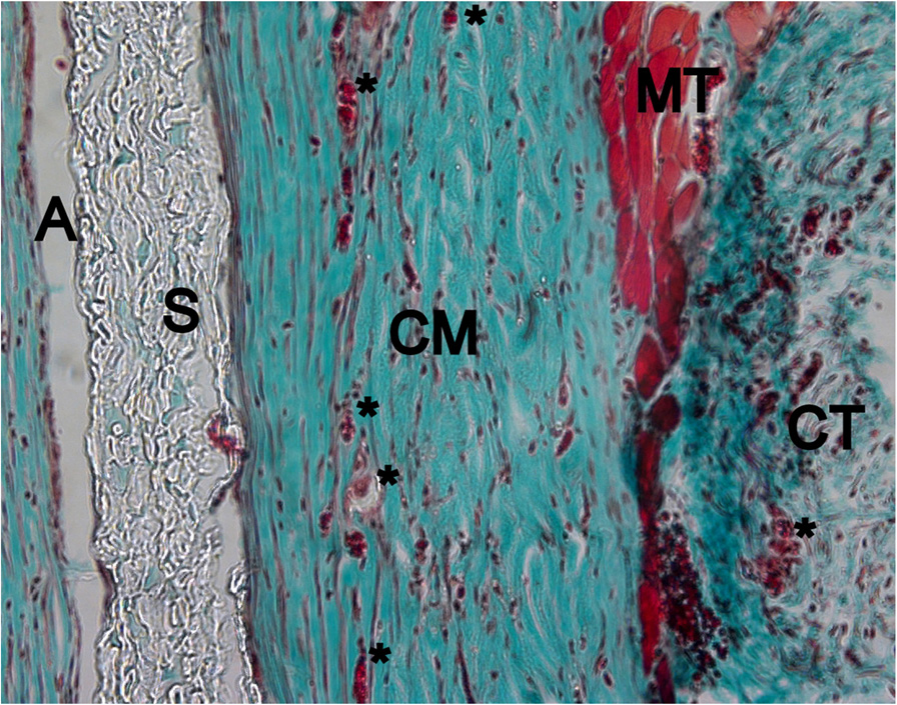
**Complete biodegradation of ND after 12 weeks healing period (Masson Goldner, Original magn. 200×).** CM: collagen matrix, MT: muscle tissue, *: blood vessel, CT: connective tissue, S: spacer, A: artefact.

**Figure 12:**
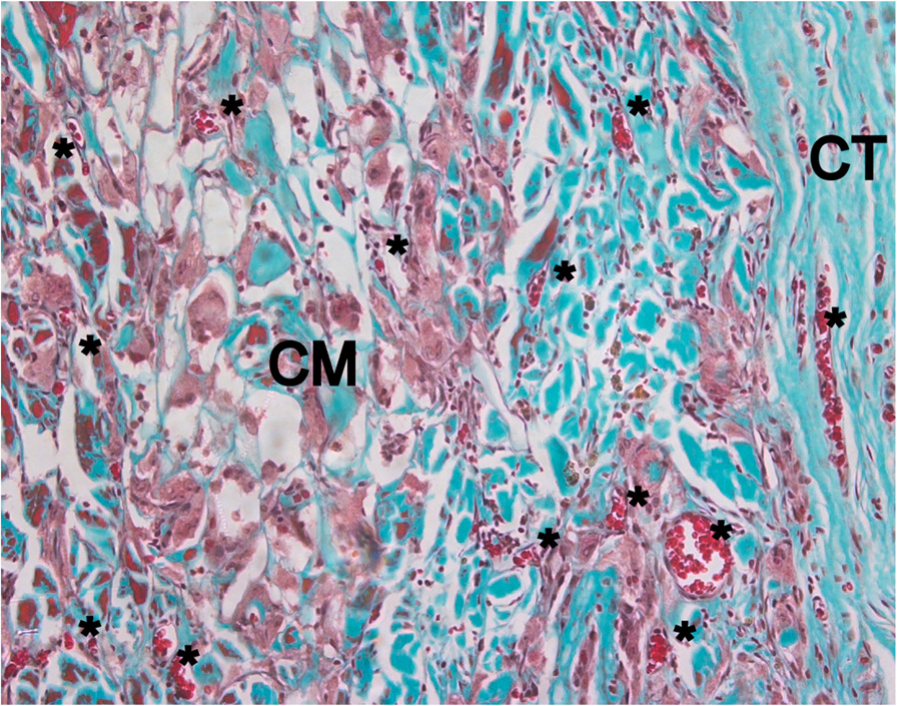
**High number of multinucleated giant cells and advanced blood vessel invasion in the ECL matrix after 12 weeks healing period (Masson Goldner, orig. magn. 200×).** CM: collagen matrix, MT: muscle tissue, *: blood vessel, CT: connective tissue.

After twelve weeks, ND, DD and DCL matrix were completely biodegraded and only a thin fibrous layer was visible between the spacer and the surrounding connective tissue (Figure 11). ECL showed only small changes in regards of membrane thickness, its collagen structure was more porous and presented some areas of fatty degeneration. The matrix was homogenously invaded blood vessels, but also a high number of multinucleated giant cells were visible inside the entire matrix body (Figure 12).

## Discussion

In the present animal study biodegradation, vascularization and tissue integration of native, differently processed and cross-linked collagen scaffolds were evaluated in a subcutaneous rat model. Within its limits, the results revealed that native, non cross-linked collagen showed a fast tissue integration and vascularization paired with slight to no signs of ingrowing inflammatory cells. Native and defatted types I and III collagens were almost completely resorbed after 12 weeks. This is in accordance to previous studies, which also found high biocompatibility and fast biodegradation for native, non cross-linked collagens, i.e. after insertion into surgical pouches of mongrel dogs, where the specimens revealed severe to moderate degradation within 4-8 weeks [26].

In contrast to native collagen, cross-linked collagen matrices have shown longer biodegradation times in previous studies. Chemical cross-linking using glutaraldehyde and enzymatic cross-linking was able to enhance the stability of collagen matrices [27]. However, it was displayed that cross-linking also influences the biological properties of collagen scaffolds. In an experimental study performed in rats different cross-linking techniques were compared to native collagen scaffolds. Membranes consisting of collagen type I, which were enzymatically cross-linked by ribose enzymes did not present any signs of biodegradation within 24 weeks and did not allow for blood vessel ingrowth or cell invasion. Glutaraldehyde cross-linking led to limited tissue integration and was accompanied by a higher degree of inflammatory response [25]. Clinically, chemical cross-linking was accompanied by more adverse events such as wound dehiscences, graft exposure and insufficient bone regeneration [28], based on the compromised tissue integration.

In the present study cross-linking using ethylene dioxide (ECL) demonstrated a longer resorption time, in line with earlier results accompanied by more inflammatory response and foreign body reaction. Tissue integration was compromised in the shorter healing periods, and multinucleated giant cells as a sign for foreign body reaction were found within the entire collagen scaffold, but thickness of the matrix scaffold remained stable during the entire study period.

But not only changed properties due to the cross-linking techniques can influence the biodegradation pattern of collagen scaffolds. Also a higher collagen density can be a reason for slower biodegradation. Scaffolds consisting of the same native collagen qualities, but differing in the density of collagen fibres, showed longer resorption times when derived from anatomically denser harvesting tissues [12]. Porous membranes offer less resistance to invading cells and blood vessels, allow for better nutrient transfer and therefore are organized faster than denser membranes [29]. ECL displayed a higher density in the histological examination and thus impeded cells from invading into the scaffolds for the first four weeks. But as the denser collagen structure might be a result of the ethylene dioxide treatment, cross-linking using ethylene dioxide seems to influence biodegradation patterns on several levels.

Defatting of collagen and dehydrothermal cross-linking did not show large differences to native collagen scaffolds. Besides comparable histomorphometrical measurements regarding membrane thickness and invasion of cells, both groups also appeared almost similar in the histological evaluation. The fast biodegradation might be a result of the collagen type I and III combined with a porous structure. Also dehydrothermally cross-linked three-dimensional collagen matrices were observed to degrade faster than chemically cross-linked scaffolds [30]. However, in contrast to defatted and native collagen, DHC displayed a slightly higher inflammatory response, hypothetically based on the thermal impact on biocompatibility of the collagen. This might be a disadvantage of dehydrothermal cross-linking.

As a drawback of the present study it has to be stated that all results were achieved in a rat model. It was demonstrated that results regarding foreign body reaction are different depending on the animal model. Mice, which were treated by a cross-linked dermal collagen, presented less signs of foreign body reaction, i.e. fewer formation of giant cells, than different strains of rats [31]. Furthermore, a clinical study that examined non cross-linked bovine type I collagen in humans revealed deviant results to previous animal studies. The membranes were intentionally exposed to the oral environment. After one week no residuals of the native membranes could be detected [32]. This is contrary to animal studies that usually found resorption times of several weeks. But the fast resorption can be explained by the presence of periodontal pathogens such as *Porphyromonas gingivalis* and *Treponema denticola*. These pathogens were observed to produce collagenases and thus promote a premature degradation of collagen [33]. However, these circumstances only apply in case of exposure to the oral cavity. As collagen matrices, in terms of soft tissue augmentation procedures, and they are ideally covered by soft tissue after flap advancement, therefore subcutaneous implantation in rats might still be an adequate model.

### Clinical relevance

The clinical gold standard in terms of soft tissue augmentation is the use of autogenous grafts harvested from the palate [15-18]. However, harvesting of these grafts is accompanied by donor site morbidity and increased stress for the patients [18,21]. Searching for non-autologous alternatives, native collagen types I and III have shown excellent biocompatibility and tissue integration in GTR and GBR procedures. Collagen types I and II of human origin have shown predictable results when used for recession coverage [34,35], increasing the width of keratinized gingiva, [36] and aesthetic soft tissue augmentations [37,38]. When a xenogeneic tissue grafting material is used, inflammation-free biodegradation is an even more important aspect. A native porcine types I and II collagen matrix revealed predictable results in enhancing the width of keratinized gingiva, reducing patient morbidity and operation time [23]. In the present study, an ethylene dioxide cross-linked matrix showed the longest volume stability, but also highest inflammatory response and foreign body reaction. Clinically, this might lead to higher rates of dehiscences and other wound healing complications as described for chemically cross-linked collagen used for GBR procedures [39]. Also a dehydrothermal cross-linking technique led to a higher inflammatory response, thus – from the clinical point of view - a native porcine collagen matrix seems to be the best option for artificial soft tissue augmentation procedures even if the volume stability is lower than with non-cross-linked collagen.

## Conclusions

Within the limits of the present study it was concluded that even if resulting in longer biodegradation times, ethylene dioxide cross-linking seems to be not suitable for stabilization of collagen used for soft tissue augmentation procedures, due to its slow tissue integration and inflammatory biodegradation pattern. Defatting using acetone and dehydrothermal cross-linking did not enhance biological properties of native types I and III collagen in terms of tissue integration or long term stability.

## Abbreviations

ND: Native Dermis; DD: Defatted Dermis; ECL: Ethylene dioxide cross-linked Dermis; DCL: Dehydrothermal cross-linked Dermis; MT: Muscle tissue; CM: Collagen matrix; CT: Connective tissue; S: Spacer; A: Artefact; *: Blood vessel; #: Multinucleated giant cell (single).

## Competing interests

The authors declare that Daniel Rothamel is a consultant and member of the supervisory board of Botiss Biomaterials, Berlin, Germany. The other authors declare that they have no competing interests.

## Authors’ contributions

DR and TF made the conception and design of the study and performed the surgical part. MB took part in the operations and performed the histological processing and evaluation, as well as the interpretation of the results and first preparation of the paper. AH, HJN and MK are co-authors of the study protocol. They were essential in obtaining the animal research committee approval and revising the paper critically for important intellectual content. JZ made a significant intellectual contribution to result interpretation and paper compilation. All authors read and approved the final manuscript.
